# Supplementary material for: SARS-CoV-2 Genomic Characteristics and Clinical Impact of SARS-CoV-2 Viral Diversity in Critically Ill COVID-19 Patients: A Prospective Multicenter Cohort Study
Source: Viruses. 2022 Jul 13;14(7):1529. doi: 10.3390/v14071529 (PMC9322524; doi:10.3390/v14071529)

SARS CoV-2 genomic characteristics and clinical impact of SARS-CoV-2 viral diversity in critically ill COVID-19 patients: a prospective multicenter cohort study

Supplementary file

Content

GISAID IDs of the sequenced samples 2

Supplementary Table S1 11

Supplementary Figure S1 12

Methods

GISAID Ids of the sequenced samples:

hCoV-19/France/IDF-HMN-20102050656/2020
hCoV-19/France/IDF-HMN-20102090001/2020
hCoV-19/France/IDF-HMN-20102110042/2020
hCoV-19/France/IDF-HMN-20102140706/2020
hCoV-19/France/IDF-HMN-20102270667/2020
hCoV-19/France/IDF-HMN-20102280010/2020
hCoV-19/France/IDF-HMN-20102290035/2020
hCoV-19/France/IDF-HMN-20112020220/2020
hCoV-19/France/IDF-HMN-20112020241/2020
hCoV-19/France/IDF-HMN-20112030383/2020
hCoV-19/France/IDF-HMN-20112040489/2020
hCoV-19/France/IDF-HMN-20112080121/2020
hCoV-19/France/IDF-HMN-20112190152/2020
hCoV-19/France/IDF-HMN-20122020179/2020
hCoV-19/France/IDF-HMN-20122070325/2020
hCoV-19/France/IDF-HMN-20122220067/2020
hCoV-19/France/IDF-HMN-20122260179/2020
hCoV-19/France/IDF-HMN-20122270023/2020
hCoV-19/France/IDF-HMN-21012150227/2021
hCoV-19/France/IDF-HMN-21012220001/2021
hCoV-19/France/IDF-HMN-21012250076/2021
hCoV-19/France/IDF-HMN-21012250077/2021
hCoV-19/France/IDF-HMN-21012250090/2021
hCoV-19/France/IDF-HMN-21012250091/2021
hCoV-19/France/IDF-HMN-21012250415/2021
hCoV-19/France/IDF-HMN-21012250669/2021
hCoV-19/France/IDF-HMN-21012300202/2021
hCoV-19/France/IDF-HMN-21012310106/2021
hCoV-19/France/IDF-HMN-21022030255/2021
hCoV-19/France/IDF-HMN-21022030571/2021
hCoV-19/France/IDF-HMN-21022050238/2021
hCoV-19/France/IDF-HMN-21022050483/2021
hCoV-19/France/IDF-HMN-21022050487/2021
hCoV-19/France/IDF-HMN-21022070154/2021
hCoV-19/France/IDF-HMN-21022080597/2021
hCoV-19/France/IDF-HMN-21022110269/2021
hCoV-19/France/IDF-HMN-21022130003/2021
hCoV-19/France/IDF-HMN-21022140003/2021
hCoV-19/France/IDF-HMN-21022140013/2021
hCoV-19/France/IDF-HMN-21022150691/2021
hCoV-19/France/IDF-HMN-21022170518/2021
hCoV-19/France/IDF-HMN-21022190564/2021

hCoV-19/France/IDF-HMN-21022200049/2021
hCoV-19/France/IDF-HMN-21022200182/2021
hCoV-19/France/IDF-HMN-21022210144/2021
hCoV-19/France/IDF-HMN-21022220043/2021
hCoV-19/France/IDF-HMN-21022220432/2021
hCoV-19/France/IDF-HMN-21022240393/2021
hCoV-19/France/IDF-HMN-21022240416/2021
hCoV-19/France/IDF-HMN-21022250001/2021
hCoV-19/France/IDF-HMN-21022250340/2021
hCoV-19/France/IDF-HMN-21022260319/2021
hCoV-19/France/IDF-HMN-21022270139/2021
hCoV-19/France/IDF-HMN-21022280188/2021
hCoV-19/France/IDF-HMN-21032010082/2021
hCoV-19/France/IDF-HMN-21032030609/2021
hCoV-19/France/IDF-HMN-21032040013/2021
hCoV-19/France/IDF-HMN-21032040454/2021
hCoV-19/France/IDF-HMN-21032040568/2021
hCoV-19/France/IDF-HMN-21032050170/2021
hCoV-19/France/IDF-HMN-21032060201/2021
hCoV-19/France/IDF-HMN-21032070125/2021
hCoV-19/France/IDF-HMN-21032080012/2021
hCoV-19/France/IDF-HMN-21032080679/2021
hCoV-19/France/IDF-HMN-21032081018/2021
hCoV-19/France/IDF-HMN-21032081084/2021
hCoV-19/France/IDF-HMN-21032090136/2021
hCoV-19/France/IDF-HMN-21032100025/2021
hCoV-19/France/IDF-HMN-21032110070/2021
hCoV-19/France/IDF-HMN-21032120565/2021
hCoV-19/France/IDF-HMN-21032120576/2021
hCoV-19/France/IDF-HMN-21032130134/2021
hCoV-19/France/IDF-HMN-21032160775/2021
hCoV-19/France/IDF-HMN-21032170105/2021
hCoV-19/France/IDF-HMN-21032200123/2021
hCoV-19/France/IDF-HMN-21032200204/2021
hCoV-19/France/IDF-HMN-21032200216/2021
hCoV-19/France/IDF-HMN-21032220978/2021
hCoV-19/France/IDF-HMN-21032230172/2021
hCoV-19/France/IDF-HMN-21032230174/2021
hCoV-19/France/IDF-HMN-21032230472/2021
hCoV-19/France/IDF-HMN-21032230511/2021
hCoV-19/France/IDF-HMN-21032231024/2021
hCoV-19/France/IDF-HMN-21032240621/2021
hCoV-19/France/IDF-HMN-21032270009/2021
hCoV-19/France/IDF-HMN-21032270182/2021
hCoV-19/France/IDF-HMN-21032290469/2021
hCoV-19/France/IDF-HMN-21032290617/2021

hCoV-19/France/IDF-HMN-21032300836/2021
hCoV-19/France/IDF-HMN-21042020139/2021
hCoV-19/France/IDF-HMN-21042040020/2021
hCoV-19/France/IDF-HMN-21042040101/2021
hCoV-19/France/IDF-HMN-21042050182/2021
hCoV-19/France/IDF-HMN-21042060234/2021
hCoV-19/France/IDF-HMN-21042061127/2021
hCoV-19/France/IDF-HMN-21042080135/2021
hCoV-19/France/IDF-HMN-21042090449/2021
hCoV-19/France/IDF-HMN-21042090934/2021
hCoV-19/France/IDF-HMN-21042110158/2021
hCoV-19/France/IDF-HMN-21042110182/2021
hCoV-19/France/IDF-HMN-21042120870/2021
hCoV-19/France/IDF-HMN-21042140013/2021
hCoV-19/France/IDF-HMN-21042160297/2021
hCoV-19/France/IDF-HMN-21042160892/2021
hCoV-19/France/IDF-HMN-21042180155/2021
hCoV-19/France/IDF-HMN-21042210512/2021
hCoV-19/France/IDF-HMN-21042210560/2021
hCoV-19/France/IDF-HMN-21042210599/2021
hCoV-19/France/IDF-HMN-21042220507/2021
hCoV-19/France/IDF-HMN-21042280540/2021
hCoV-19/France/IDF-HMN-21042280543/2021
hCoV-19/France/IDF-HMN-21052040088/2021
hCoV-19/France/IDF-HMN-21052070581/2021
hCoV-19/France/IDF-HMN-21052140053/2021
hCoV-19/France/IDF-HMN-21052140055/2021
hCoV-19/France/IDF-HMN-21052140567/2021
hCoV-19/France/IDF-HMN-21052150032/2021
hCoV-19/France/IDF-HMN-21052160070/2021
hCoV-19/France/IDF-HMN-21052170806/2021
hCoV-19/France/IDF-HMN-21052190275/2021
hCoV-19/France/IDF-HMN-21052190280/2021
hCoV-19/France/IDF-HMN-21052190283/2021
hCoV-19/France/IDF-HMN-21052190284/2021
hCoV-19/France/IDF-HMN-21052190286/2021
hCoV-19/France/IDF-HMN-21052190287/2021
hCoV-19/France/IDF-HMN-21052190288/2021
hCoV-19/France/IDF-HMN-21052190289/2021
hCoV-19/France/IDF-HMN-21052190290/2021
hCoV-19/France/IDF-HMN-21052190293/2021
hCoV-19/France/IDF-HMN-21052190295/2021
hCoV-19/France/IDF-HMN-21052190298/2021
hCoV-19/France/IDF-HMN-21052190301/2021
hCoV-19/France/IDF-HMN-21052190305/2021
hCoV-19/France/IDF-HMN-21052190309/2021

hCoV-19/France/IDF-HMN-21052190312/2021
hCoV-19/France/IDF-HMN-21052190313/2021
hCoV-19/France/IDF-HMN-21052190314/2021
hCoV-19/France/IDF-HMN-21052190316/2021
hCoV-19/France/IDF-HMN-21052190317/2021
hCoV-19/France/IDF-HMN-21052190318/2021
hCoV-19/France/IDF-HMN-21052190319/2021
hCoV-19/France/IDF-HMN-21052190320/2021
hCoV-19/France/IDF-HMN-21052190322/2021
hCoV-19/France/IDF-HMN-21052190324/2021
hCoV-19/France/IDF-HMN-21052190326/2021
hCoV-19/France/IDF-HMN-21052190347/2021
hCoV-19/France/IDF-HMN-21052190349/2021
hCoV-19/France/IDF-HMN-21052190350/2021
hCoV-19/France/IDF-HMN-21052190351/2021
hCoV-19/France/IDF-HMN-21052190352/2021
hCoV-19/France/IDF-HMN-21052190353/2021
hCoV-19/France/IDF-HMN-21052190418/2021
hCoV-19/France/IDF-HMN-21052190419/2021
hCoV-19/France/IDF-HMN-21052190421/2021
hCoV-19/France/IDF-HMN-21052190423/2021
hCoV-19/France/IDF-HMN-21052190426/2021
hCoV-19/France/IDF-HMN-21052190430/2021
hCoV-19/France/IDF-HMN-21052190442/2021
hCoV-19/France/IDF-HMN-21052190445/2021
hCoV-19/France/IDF-HMN-21052190462/2021
hCoV-19/France/IDF-HMN-21052190463/2021
hCoV-19/France/IDF-HMN-21052190466/2021
hCoV-19/France/IDF-HMN-21052190469/2021
hCoV-19/France/IDF-HMN-21052190472/2021
hCoV-19/France/IDF-HMN-21052190474/2021
hCoV-19/France/IDF-HMN-21052190480/2021
hCoV-19/France/IDF-HMN-21052190482/2021
hCoV-19/France/IDF-HMN-21052190485/2021
hCoV-19/France/IDF-HMN-21052190488/2021
hCoV-19/France/IDF-HMN-21052230088/2021
hCoV-19/France/IDF-HMN-21052270599/2021
hCoV-19/France/IDF-HMN-21052270601/2021
hCoV-19/France/IDF-HMN-21052270603/2021
hCoV-19/France/IDF-HMN-21052270604/2021
hCoV-19/France/IDF-HMN-21052270605/2021
hCoV-19/France/IDF-HMN-21052270607/2021
hCoV-19/France/IDF-HMN-21052270608/2021
hCoV-19/France/IDF-HMN-21052270610/2021
hCoV-19/France/IDF-HMN-21052270618/2021
hCoV-19/France/IDF-HMN-21052270625/2021

hCoV-19/France/IDF-HMN-21052270626/2021
hCoV-19/France/IDF-HMN-21052270627/2021
hCoV-19/France/IDF-HMN-21052270629/2021
hCoV-19/France/IDF-HMN-21052270630/2021
hCoV-19/France/IDF-HMN-21052270631/2021
hCoV-19/France/IDF-HMN-21052270638/2021
hCoV-19/France/IDF-HMN-21052270646/2021
hCoV-19/France/IDF-HMN-21052270647/2021
hCoV-19/France/IDF-HMN-21052270649/2021
hCoV-19/France/IDF-HMN-21052270652/2021
hCoV-19/France/IDF-HMN-21052270654/2021
hCoV-19/France/IDF-HMN-21052270657/2021
hCoV-19/France/IDF-HMN-21052270675/2021
hCoV-19/France/IDF-HMN-21052270676/2021
hCoV-19/France/IDF-HMN-21052270678/2021
hCoV-19/France/IDF-HMN-21052270679/2021
hCoV-19/France/IDF-HMN-21052270680/2021
hCoV-19/France/IDF-HMN-21052270684/2021
hCoV-19/France/IDF-HMN-21052270686/2021
hCoV-19/France/IDF-HMN-21052270688/2021
hCoV-19/France/IDF-HMN-21052270694/2021
hCoV-19/France/IDF-HMN-21052270699/2021
hCoV-19/France/IDF-HMN-21052270701/2021
hCoV-19/France/IDF-HMN-21052270707/2021
hCoV-19/France/IDF-HMN-21052270712/2021
hCoV-19/France/IDF-HMN-21052270714/2021
hCoV-19/France/IDF-HMN-21052270716/2021
hCoV-19/France/IDF-HMN-21052270719/2021
hCoV-19/France/IDF-HMN-21052270734/2021
hCoV-19/France/IDF-HMN-21052270737/2021
hCoV-19/France/IDF-HMN-21052270739/2021
hCoV-19/France/IDF-HMN-21052280096/2021
hCoV-19/France/IDF-HMN-21052280105/2021
hCoV-19/France/IDF-HMN-21052280121/2021
hCoV-19/France/IDF-HMN-21052280123/2021
hCoV-19/France/IDF-HMN-21052280126/2021
hCoV-19/France/IDF-HMN-21052280127/2021
hCoV-19/France/IDF-HMN-21052280129/2021
hCoV-19/France/IDF-HMN-21052280130/2021
hCoV-19/France/IDF-HMN-21052280131/2021
hCoV-19/France/IDF-HMN-21052280133/2021
hCoV-19/France/IDF-HMN-21052280134/2021
hCoV-19/France/IDF-HMN-21052280135/2021
hCoV-19/France/IDF-HMN-21052280136/2021
hCoV-19/France/IDF-HMN-21052280138/2021
hCoV-19/France/IDF-HMN-21052280139/2021

hCoV-19/France/IDF-HMN-21052280140/2021
hCoV-19/France/IDF-HMN-21052280141/2021
hCoV-19/France/IDF-HMN-21052280143/2021
hCoV-19/France/IDF-HMN-21052280145/2021
hCoV-19/France/IDF-HMN-21052280147/2021
hCoV-19/France/IDF-HMN-21052280148/2021
hCoV-19/France/IDF-HMN-21052280150/2021
hCoV-19/France/IDF-HMN-21052280153/2021
hCoV-19/France/IDF-HMN-21052280155/2021
hCoV-19/France/IDF-HMN-21052280157/2021
hCoV-19/France/IDF-HMN-21052280158/2021
hCoV-19/France/IDF-HMN-21052280164/2021
hCoV-19/France/IDF-HMN-21052280165/2021
hCoV-19/France/IDF-HMN-21052280173/2021
hCoV-19/France/IDF-HMN-21052280177/2021
hCoV-19/France/IDF-HMN-21052280178/2021
hCoV-19/France/IDF-HMN-21052280197/2021
hCoV-19/France/IDF-HMN-21052280199/2021
hCoV-19/France/IDF-HMN-21052280203/2021
hCoV-19/France/IDF-HMN-21052280222/2021
hCoV-19/France/IDF-HMN-21052280223/2021
hCoV-19/France/IDF-HMN-21052280224/2021
hCoV-19/France/IDF-HMN-21052280225/2021
hCoV-19/France/IDF-HMN-21052280226/2021
hCoV-19/France/IDF-HMN-21052280227/2021
hCoV-19/France/IDF-HMN-21052280229/2021
hCoV-19/France/IDF-HMN-21052280230/2021
hCoV-19/France/IDF-HMN-21052280241/2021
hCoV-19/France/IDF-HMN-21052280242/2021
hCoV-19/France/IDF-HMN-21052280244/2021
hCoV-19/France/IDF-HMN-21052280245/2021
hCoV-19/France/IDF-HMN-21052280246/2021
hCoV-19/France/IDF-HMN-21052280247/2021
hCoV-19/France/IDF-HMN-21052280248/2021
hCoV-19/France/IDF-HMN-21052280249/2021
hCoV-19/France/IDF-HMN-21052280251/2021
hCoV-19/France/IDF-HMN-21052280253/2021
hCoV-19/France/IDF-HMN-21052280255/2021
hCoV-19/France/IDF-HMN-21052280256/2021
hCoV-19/France/IDF-HMN-21052280259/2021
hCoV-19/France/IDF-HMN-21052280260/2021
hCoV-19/France/IDF-HMN-21052280261/2021
hCoV-19/France/IDF-HMN-21052280262/2021
hCoV-19/France/IDF-HMN-21052280263/2021
hCoV-19/France/IDF-HMN-21052280285/2021
hCoV-19/France/IDF-HMN-21052280302/2021

hCoV-19/France/IDF-HMN-21052280304/2021
hCoV-19/France/IDF-HMN-21052280305/2021
hCoV-19/France/IDF-HMN-21052280306/2021
hCoV-19/France/IDF-HMN-21052280322/2021
hCoV-19/France/IDF-HMN-21052280323/2021
hCoV-19/France/IDF-HMN-21052280324/2021
hCoV-19/France/IDF-HMN-21052280524/2021
hCoV-19/France/IDF-HMN-21052310450/2021
hCoV-19/France/IDF-HMN-21052310451/2021
hCoV-19/France/IDF-HMN-21052310452/2021
hCoV-19/France/IDF-HMN-21052310454/2021
hCoV-19/France/IDF-HMN-21052310459/2021
hCoV-19/France/IDF-HMN-21052310460/2021
hCoV-19/France/IDF-HMN-21052310461/2021
hCoV-19/France/IDF-HMN-21052310462/2021
hCoV-19/France/IDF-HMN-21052310463/2021
hCoV-19/France/IDF-HMN-21052310466/2021
hCoV-19/France/IDF-HMN-21052310467/2021
hCoV-19/France/IDF-HMN-21052310468/2021
hCoV-19/France/IDF-HMN-21052310470/2021
hCoV-19/France/IDF-HMN-21052310475/2021
hCoV-19/France/IDF-HMN-21052310477/2021
hCoV-19/France/IDF-HMN-21052310479/2021
hCoV-19/France/IDF-HMN-21062030211/2021
hCoV-19/France/IDF-HMN-21062030259/2021
hCoV-19/France/IDF-HMN-21062030263/2021
hCoV-19/France/IDF-HMN-21062030271/2021
hCoV-19/France/IDF-HMN-21062030274/2021
hCoV-19/France/IDF-HMN-21062030275/2021
hCoV-19/France/IDF-HMN-21062030277/2021
hCoV-19/France/IDF-HMN-21062030278/2021
hCoV-19/France/IDF-HMN-21062030296/2021
hCoV-19/France/IDF-HMN-21062030304/2021
hCoV-19/France/IDF-HMN-21062030316/2021
hCoV-19/France/IDF-HMN-21062030317/2021
hCoV-19/France/IDF-HMN-21062030319/2021
hCoV-19/France/IDF-HMN-21062030328/2021
hCoV-19/France/IDF-HMN-21062030358/2021
hCoV-19/France/IDF-HMN-21062030359/2021
hCoV-19/France/IDF-HMN-21062030486/2021
hCoV-19/France/IDF-HMN-21062030488/2021
hCoV-19/France/IDF-HMN-21062030492/2021
hCoV-19/France/IDF-HMN-21062030494/2021
hCoV-19/France/IDF-HMN-21062030496/2021
hCoV-19/France/IDF-HMN-21062030497/2021
hCoV-19/France/IDF-HMN-21062030500/2021

hCoV-19/France/IDF-HMN-21062040156/2021
hCoV-19/France/IDF-HMN-21062040157/2021
hCoV-19/France/IDF-HMN-21062040161/2021
hCoV-19/France/IDF-HMN-21062040164/2021
hCoV-19/France/IDF-HMN-21062040165/2021
hCoV-19/France/IDF-HMN-21062040172/2021
hCoV-19/France/IDF-HMN-21062040179/2021
hCoV-19/France/IDF-HMN-21062040203/2021
hCoV-19/France/IDF-HMN-21062040206/2021
hCoV-19/France/IDF-HMN-21062040207/2021
hCoV-19/France/IDF-HMN-21062040208/2021
hCoV-19/France/IDF-HMN-21062040209/2021
hCoV-19/France/IDF-HMN-21062040210/2021
hCoV-19/France/IDF-HMN-21062040216/2021
hCoV-19/France/IDF-HMN-21062040218/2021
hCoV-19/France/IDF-HMN-21062040248/2021
hCoV-19/France/IDF-HMN-21062040249/2021
hCoV-19/France/IDF-HMN-21062040255/2021
hCoV-19/France/IDF-HMN-21062040256/2021
hCoV-19/France/IDF-HMN-21062040258/2021
hCoV-19/France/IDF-HMN-21062040267/2021
hCoV-19/France/IDF-HMN-21062040273/2021
hCoV-19/France/IDF-HMN-21062040278/2021
hCoV-19/France/IDF-HMN-21062040281/2021
hCoV-19/France/IDF-HMN-21062040286/2021
hCoV-19/France/IDF-HMN-21062040289/2021
hCoV-19/France/IDF-HMN-21062080280/2021
hCoV-19/France/IDF-HMN-21062080282/2021
hCoV-19/France/IDF-HMN-21062080283/2021
hCoV-19/France/IDF-HMN-21062080284/2021
hCoV-19/France/IDF-HMN-21062080356/2021
hCoV-19/France/IDF-HMN-21062080367/2021
hCoV-19/France/IDF-HMN-21062080369/2021
hCoV-19/France/IDF-HMN-21062080372/2021
hCoV-19/France/IDF-HMN-21062080376/2021
hCoV-19/France/IDF-HMN-21062080378/2021
hCoV-19/France/IDF-HMN-21062080383/2021
hCoV-19/France/IDF-HMN-21062080384/2021
hCoV-19/France/IDF-HMN-21062080387/2021
hCoV-19/France/IDF-HMN-21062080389/2021
hCoV-19/France/IDF-HMN-21062080392/2021
hCoV-19/France/IDF-HMN-21062080397/2021
hCoV-19/France/IDF-HMN-21062090072/2021
hCoV-19/France/IDF-HMN-21062090290/2021
hCoV-19/France/IDF-HMN-21062090292/2021
hCoV-19/France/IDF-HMN-21062090293/2021

hCoV-19/France/IDF-HMN-21062090295/2021
hCoV-19/France/IDF-HMN-21062090297/2021
hCoV-19/France/IDF-HMN-21062090300/2021
hCoV-19/France/IDF-HMN-21062090301/2021
hCoV-19/France/IDF-HMN-21062090303/2021
hCoV-19/France/IDF-HMN-21062090304/2021
hCoV-19/France/IDF-HMN-21062090305/2021
hCoV-19/France/IDF-HMN-21062090306/2021
hCoV-19/France/IDF-HMN-21062090307/2021
hCoV-19/France/IDF-HMN-21062090308/2021
hCoV-19/France/IDF-HMN-21062090309/2021
hCoV-19/France/IDF-HMN-21062090310/2021
hCoV-19/France/IDF-HMN-21062090311/2021
hCoV-19/France/IDF-HMN-21062090312/2021
hCoV-19/France/IDF-HMN-21062090313/2021
hCoV-19/France/IDF-HMN-21062090314/2021
hCoV-19/France/IDF-HMN-21062090315/2021
hCoV-19/France/IDF-HMN-21062090316/2021
hCoV-19/France/IDF-HMN-21062090317/2021
hCoV-19/France/IDF-HMN-21062090318/2021
hCoV-19/France/IDF-HMN-21062090319/2021
hCoV-19/France/IDF-HMN-21062090516/2021

							2020																		2021									
	S40	S41	S42	S43	S44	S45	S46	S47	S49	S50	S51	S52	S53	S1	S2	S3	S4	S5	S6	S7	S8	S9	S10	S11	S12	S13	S14	S15	S16	S17	S18	S19	S20	S21
**α (B.1.1.7)**												1			2	5	6	14	16	11	22	23	24	29	13	5	4	6	4	1	2	3	3	1
**β (B.1.351)**																		2	3		3	3	3		1		2		1			1		
**γ (P.1)**																		1		1				1										
**B.1.474**				1														1																
**B.1.525**																							1			1	1							
**C.16**																			1			1												
**C.35**																								1										
**C.36**																			1															
**A21**																		3		1	1													
**A27**																			1		1			1										
**B.1.1**				1																														
**B.1.222**																						1												
**B.1.1.241**			1	1	2		1			1			1			1																		
**B.1.1.269**									1																									
**B.1.317**			1												1																			
**B.1.1.420**																				1														
**B.1.1.50**		1																																
**B.1.1.519**																	1																	
**B.1.2**							1	1				1			1																			
**B.1.210**												1																						
**B.1.214**																				1	1		1											
**B.1.221**				1												1																		
**B.1.258**						1																												
**B.1.36**					1																													
**B.1.416.1**	1																																	
**B.1.177**			4	2	3	6	3	2	1	1	1		5	1	4		1			1	1		1											
**B.1**	1	1	2	1	1	2	1		3			1					1			1														
**B.1.160**	3	6	4	5	15	6	4	7	1	5	4	5	3			5	4	6	8		2		1											

Supplementary Table S1: Time course of emerging SARS-CoV-2 variants during the study period. Number of samples corresponding to each variant are shown in each week of the study period.

Supplementary Figure S1. Phylogenetic tree performed after full-length genome alignment. The phylogenetic tree shows that the mutation Orf3a-Q57H is mainly harbored by variants from lineages B.1.160 and B.1.351.

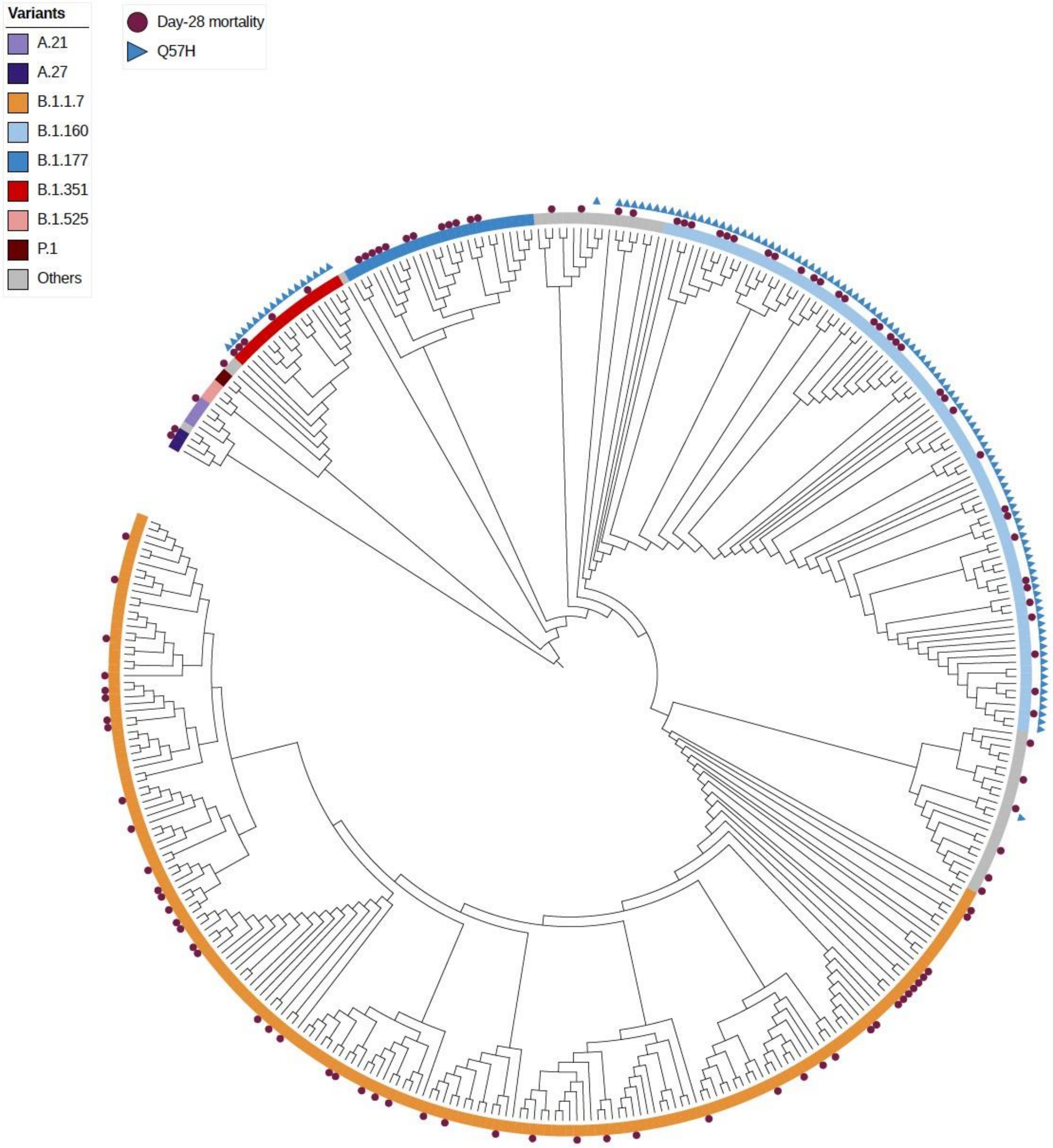

Supplement: Supplementary file 1 [file viruses-14-01529-s001.zip › viruses-1748083-supplementary.pdf]
